# Supplementary material for: Self-assembling human heart organoids for the modeling of cardiac development and congenital heart disease
Source: Nat Commun. 2021 Aug 26;12:5142. doi: 10.1038/s41467-021-25329-5 (PMC8390749; doi:10.1038/s41467-021-25329-5)
Supplement: Supplementary file 1 — Supplementary information [file 41467_2021_25329_MOESM1_ESM.pdf]

**SUPPLEMENTARY INFORMATION**

# SELF-ASSEMBLING HUMAN HEART ORGANOIDS FOR THE MODELING OF CARDIAC DEVELOPMENT AND CONGENITAL HEART DISEASE

Yonatan R. Lewis--Israeli[1,2], Aaron H. Wasserman[1,2], Mitchell A. Gabalski[1,2], Brett D. Volmert[1,2], Yixuan Ming[3], Kristen A. Ball[1,2], Weiyang Yang[4,5], Jinyun Zou[3], Guangming Ni[3], Natalia Pajares[6], Xanthippi Chatzistavrou[6], Wen Li[4,5], Chao Zhou[3] and Aitor Aguirre[1,2*]

[1]Division of Developmental and Stem Cell Biology, Institute for Quantitative Health Science and Engineering, Michigan State University, MI, USA

[2]Department of Biomedical Engineering, College of Engineering, Michigan State University, MI, USA

[3]Department of Biomedical Engineering, Washington University in Saint Louis, MO, USA

[4]Division of Biomedical Devices, Institute for Quantitative Health Science and Engineering, Michigan State University, MI, USA

[5]Department of Electrical and Computer Engineering, College of Engineering, Michigan State University, MI, USA

[6]Department of Chemical Engineering and Material Science, College of Engineering, Michigan State University, MI, USA

*Corresponding author: Aitor Aguirre (email: aaguirre@msu.edu)

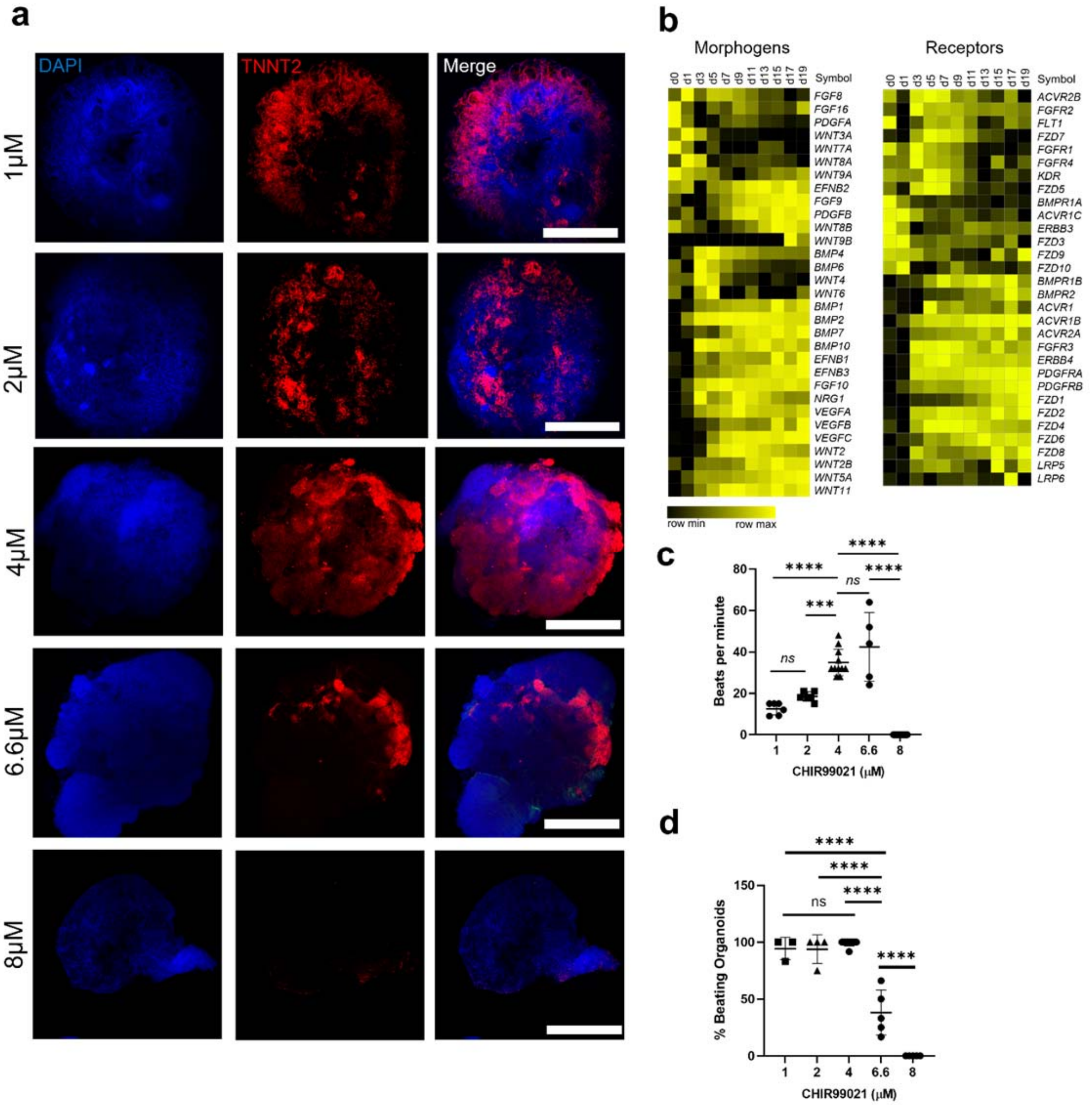


**Supplementary Figure 1. a,** Confocal immunofluorescent images for DAPI (blue) and TNNT2 (red), in organoids with CHIR99021 exposure concentrations of 1, 2, 4, 6.6, and 8 μM, at day 15; scale bars, 500μm (n=12). **b**, Morphogen and receptor expression levels as determined by RNA-seq heatmaps in differentiating hHOs from day 0 to day 19. **c,** Quantification of beat frequency in hHOs (n=6) and **d**, percentage of beating hHOs per treatment (1 μM n=288 organoids, 2 μM n=384 organoids, 4 μM n=960, 6.6 and 8 μM n=480 organoids). Value = mean ± s.d., 1-way ANOVA multiple comparison test; ***p=0.0003, ****p<0.0001, otherwise ns: no significance. Heatmap colors are relative intensity representing gene expression. Source data are provided as a Source Data file.

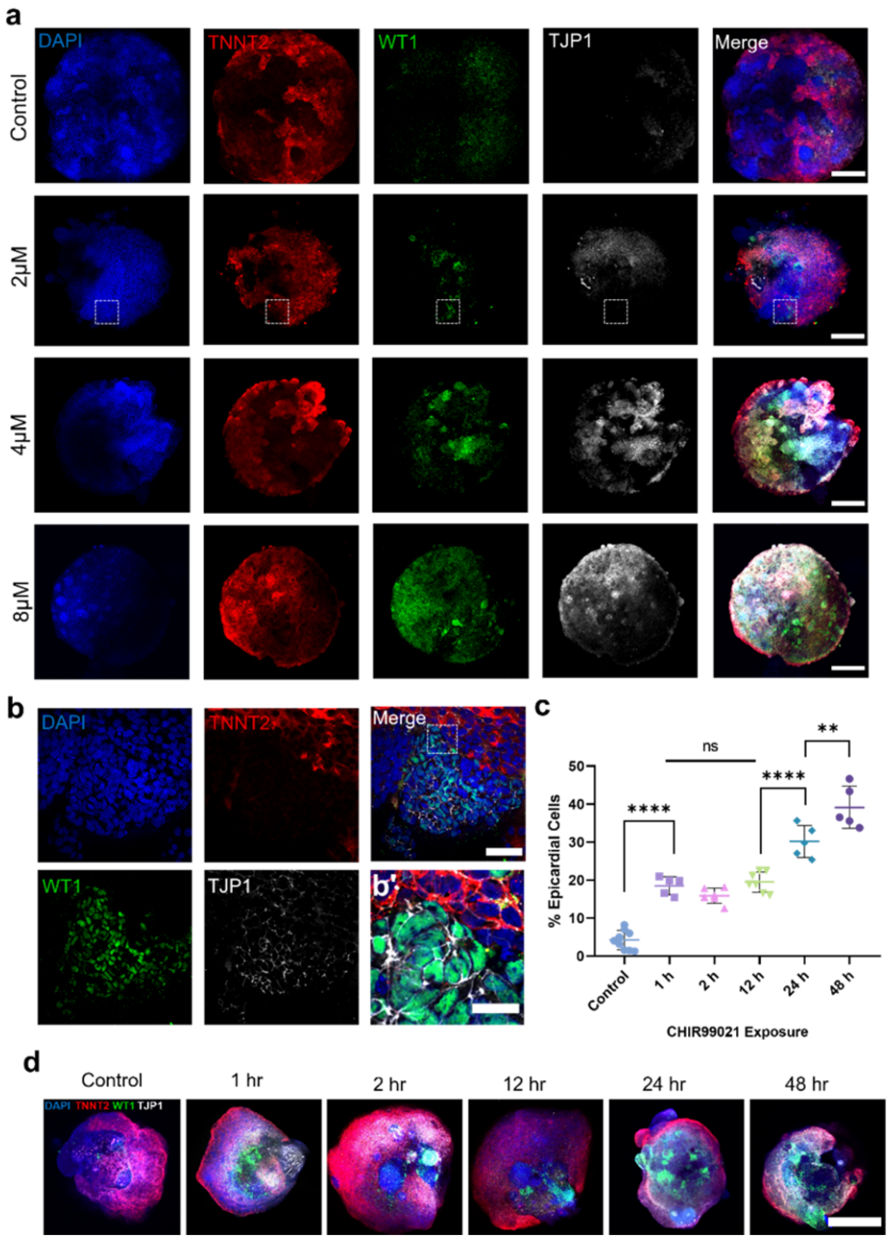


**Supplementary Figure 2. a,** Confocal immunofluorescent images of hHOs at differentiation day 15 for DAPI (blue), WT1 (green), TNNT2 (red), and TJP1 (white), with variable concentrations of the second CHIR exposure at day 7 vs. control with no second CHIR exposure; scale bars: 500 µm, and **b**, high magnification of hHOs sfter 2 µM second CHIR exposure showing an adjacent region of TNNT2$^+$ myocardial tissue and WT1$^+$/TJP1$^+$ epicardial tissue; scale bar: 50 µm. **c,** Cell quantification analysis of cardiomyocyte (TNNT2$^+$) and epicardial cells (WT1$^+$ and TJP1$^+$) within organoids taken at multiple z-planes as a percentage of DAPI$^+$ cells of each organoid treated with CHIR99021 at day 7 for different time durations (control n=9 organoids, 1, 24 and 48 hours n=5 organoids, 2 hours n=6 organoids, 12 hours n = 7 organoids), and **d,** representative confocal immunofluorescent images of organoids from these time durations; scale bar: 500 µm. Value = mean ± s.d., 1-way ANOVA multiple comparison test; **p=0.0018, ****p<0.0001, otherwise ns: no significance. Source data are provided as a Source Data file.

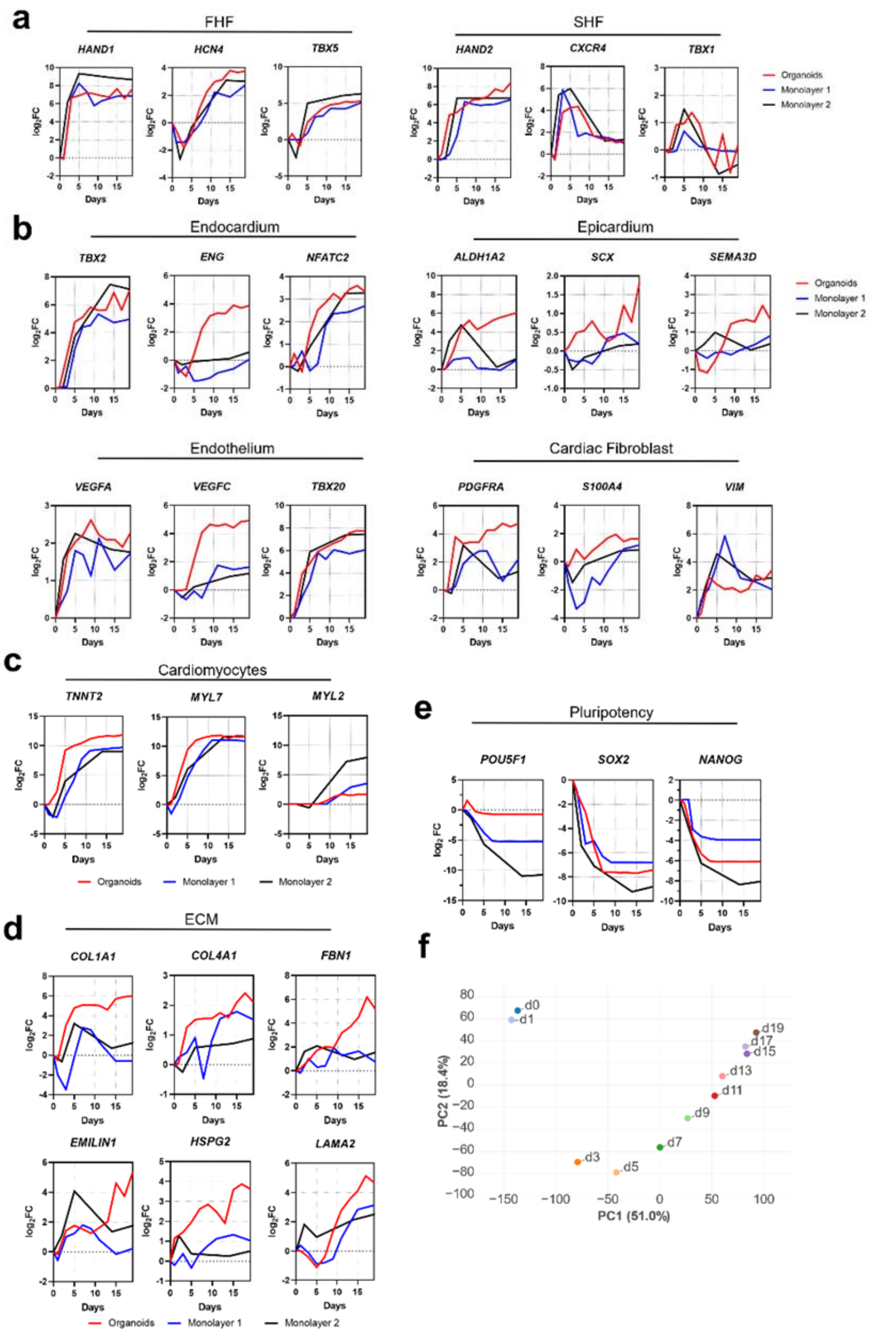


**Supplementary Figure 3. a,** Gene expression analysis of additional first and second heart field markers over heart organoid differentiation. **b–f**, Gene expression analysis ($log_2$ fold-change vs. D0) for **b,** cardiac-specific cell type populations in heart organoids, including (from top left to bottom right) endocardial cells, epicardial cells, endothelium, and cardiac fibroblasts. **c**, cardiomyocyte markers, **d**, ECM protein-coding genes that are present in cardiac tissue, and **e**, for pluripotency markers. **f**, Principal component analysis of heart organoid differentiation over time. ECM: extracellular matrix, FHF: first heart field, PC: principal component, SHF: second heart field.

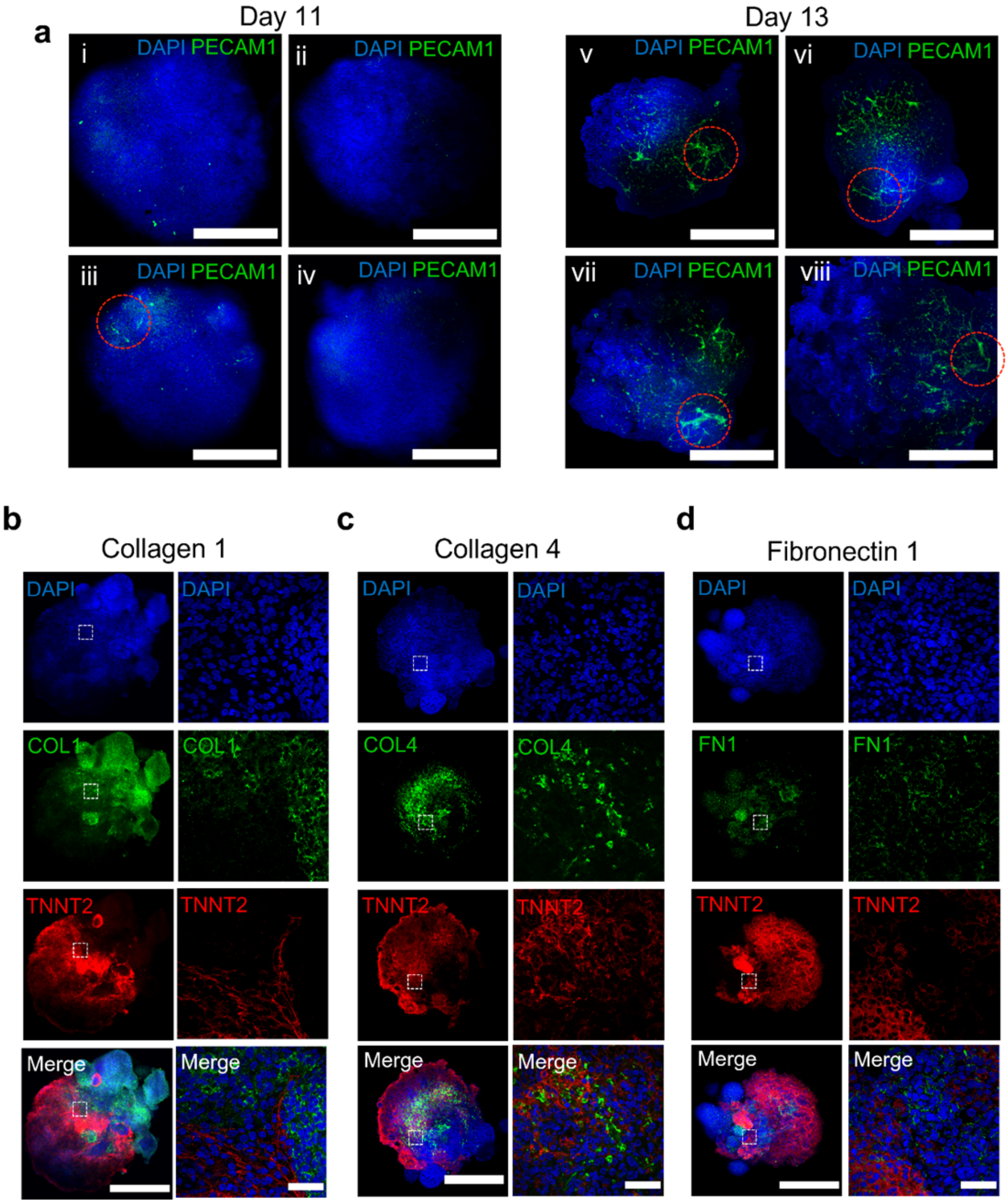


**Supplementary Figure 4.** Confocal immunofluorescent images of hHOs, DAPI in blue and TNNT2 in red. **a**, Day 11 and day 13 organoids showing maximum intensity projections of PECAM1 (green) endothelial cells forming robust vascular-like network throughout the hHOs; red dotted circles indicate region of high vascular branching. **b–d**, Presence of ECM proteins in green in day 15 organoids, showing collagen 1 (**b**), collagen 4 (**c**), and fibronectin 1 (**d**); in green; scale bars: 500 µm, inset: 50 µm.

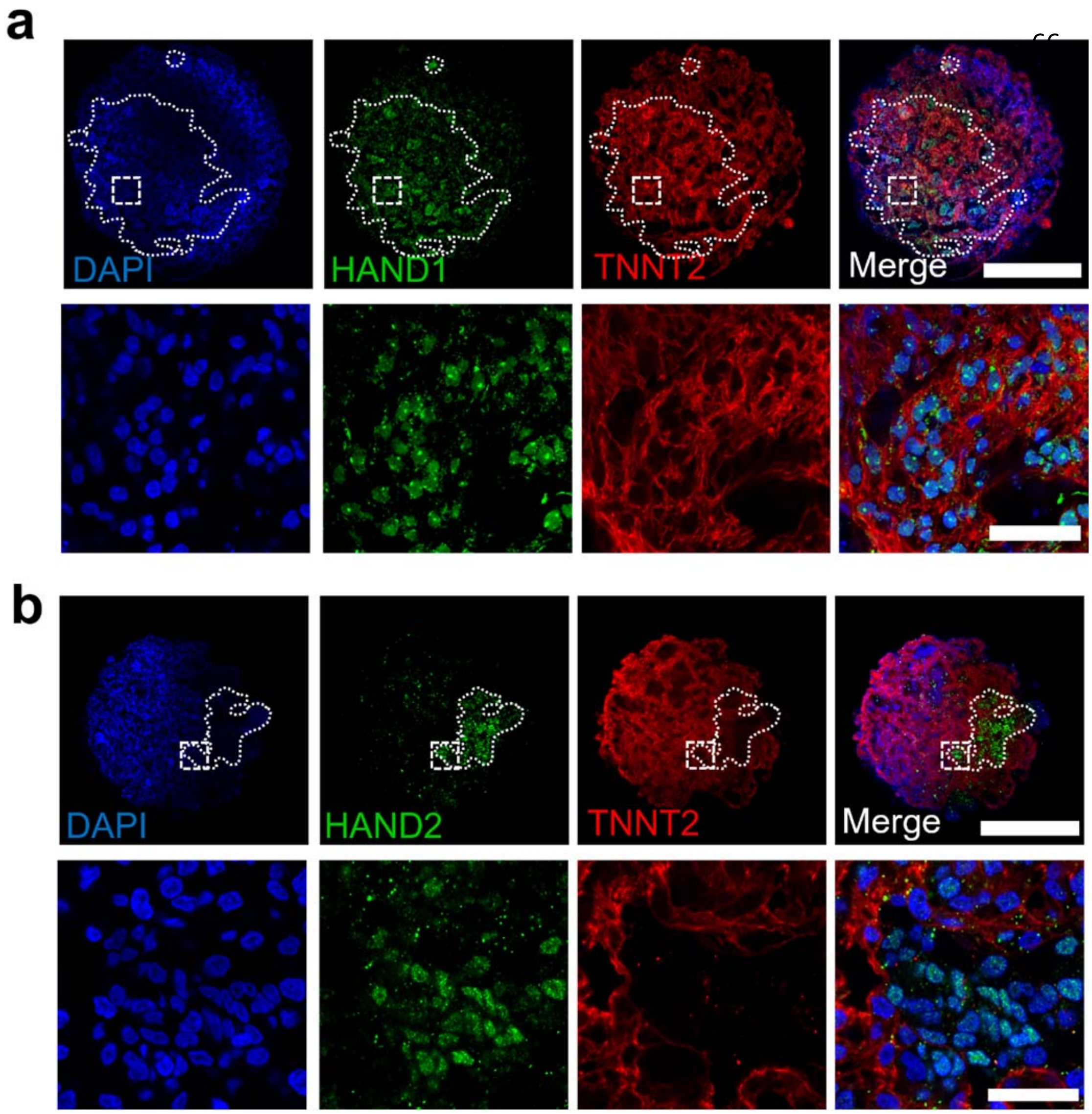


**Supplementary Figure 5. a**-**b,** Confocal immunofluorescent images of hHOs at differentiation day 8. **a**, FHF marker HAND1 (green) in defined area of the hHO, and **b**, SHF marker HAND2 (green); DAPI (blue), TNNT2 (red); scale bars: 500 µm, inset: 50 µm.

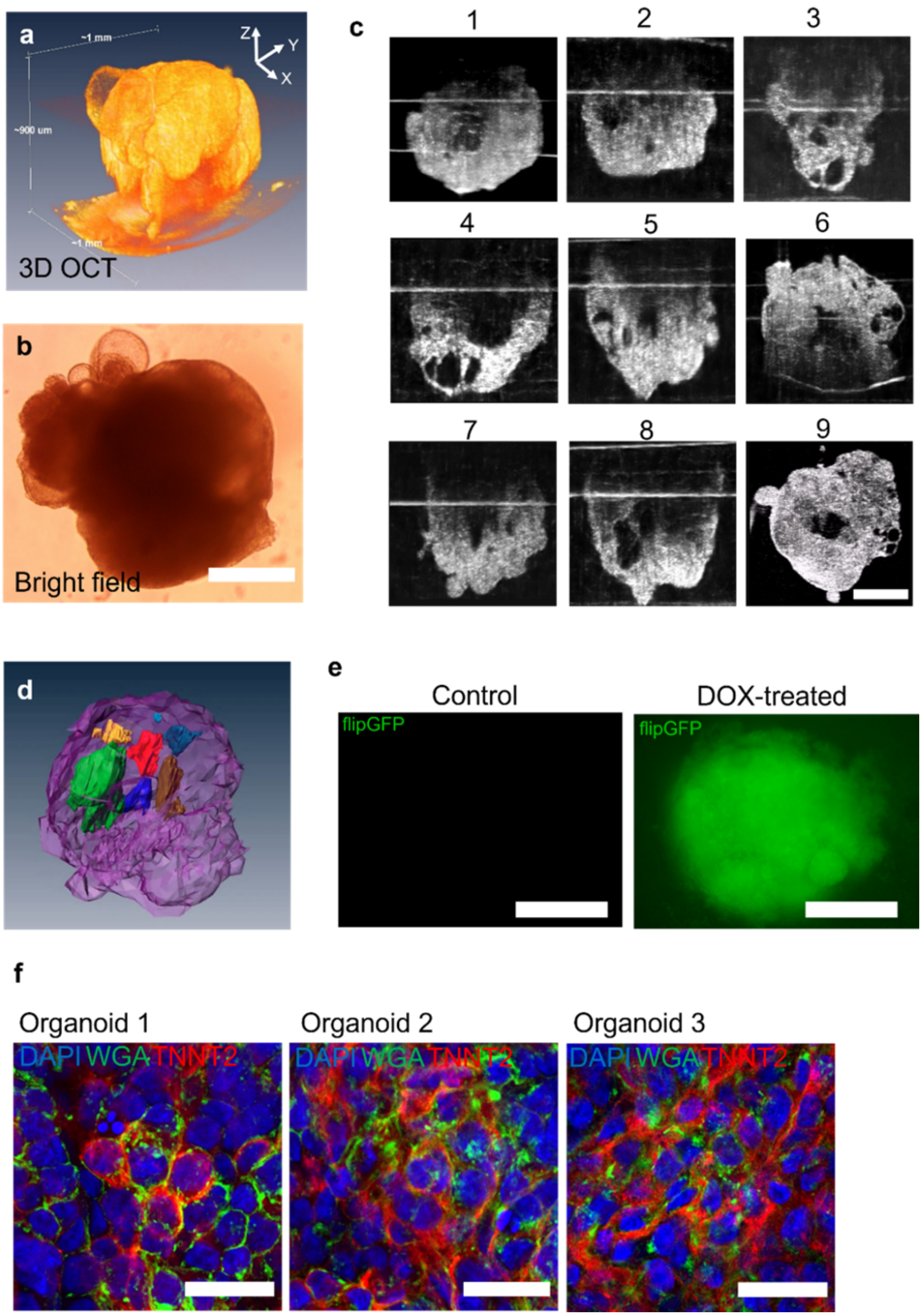


**Supplementary Figure 6. a,** 3D reconstruction of OCT images and **b,** bright field image of hHO. **c**, OCT images showing cross-sections of the center of 9 different organoids, revealing central chambers; scale bar: 500 µm. **d**, 3D reconstruction of chambers observed via OCT imaging. **e**, Immunofluorescence images of organoids derived from a FlipGFP transgenic iPSC line L1 showing no apoptosis in control hHOs (left) and high apoptosis in hHOs treated with 5 µM Doxorubicin (DOX) (right); scale bar: 500µm. **f**, Representative immunofluorescence imaging of t-tubules-like structures in day 15 hHOs, using WGA (green) as a t-tubule indicator; scale bar: 50 µm.

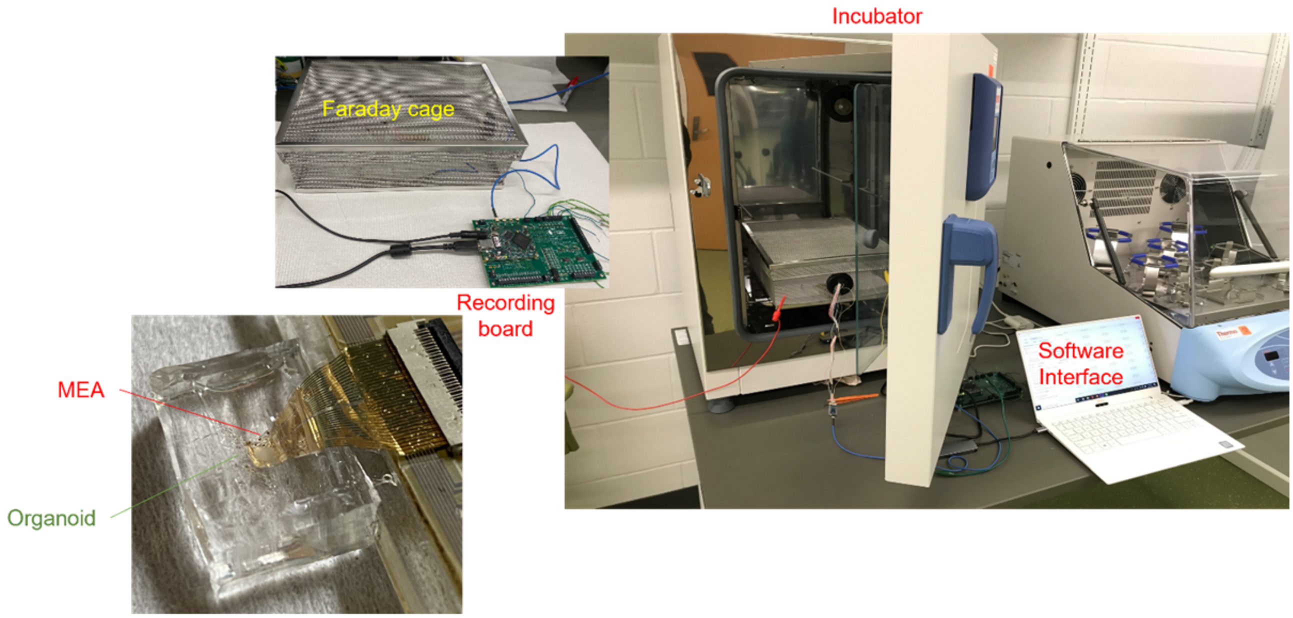


**Supplementary Figure 7.** Microelectrode array (MEA) recording system showing the gold electrode array in a PDMS chamber where the organoid is placed within a Faraday cage inside an incubator.

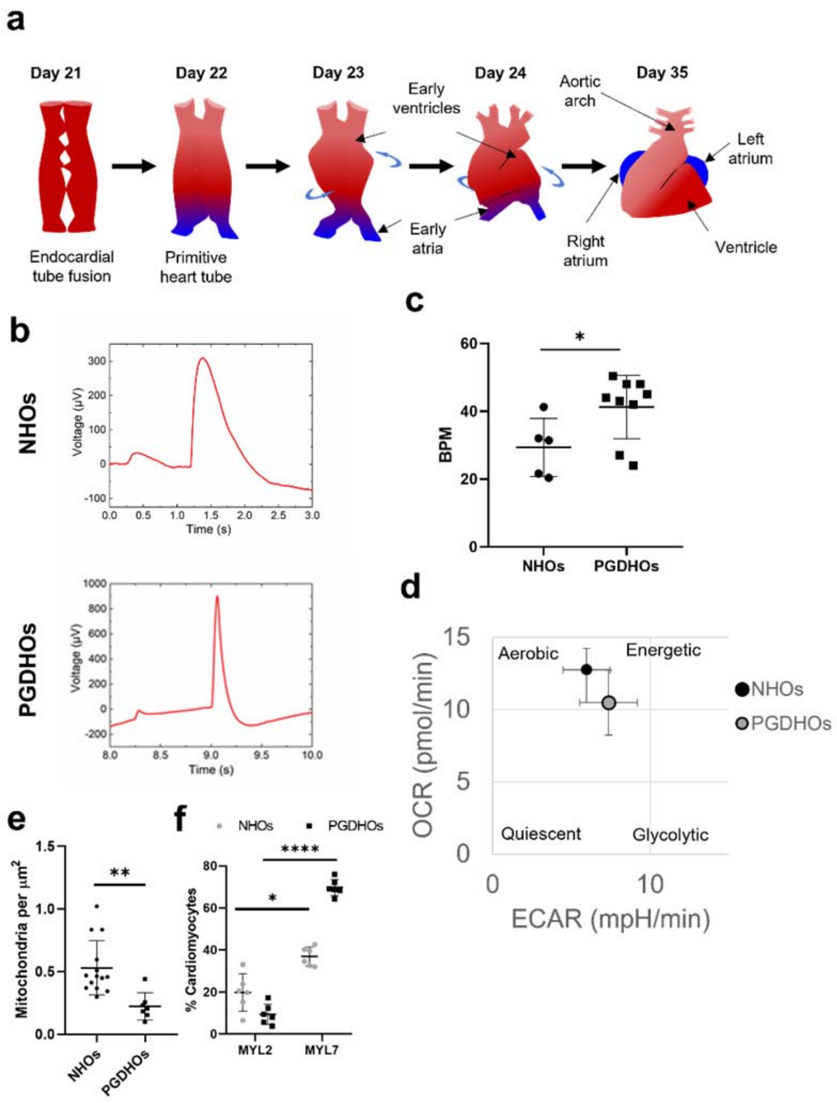


**Supplementary Figure 8. Human heart organoids modeling functional features healthy vs. diabetic conditions. a,** Schematic of heart tube formation and looping into the four chambers of the heart. **b,** Representative MEA electrophysiology detail of normal vs. diabetic organoids. **c,** beating frequency in beats per minute (BPM) in normal and diabetic organoids as recorded by MEA (value = mean ± s.d, NHOs n=5 organoids, PGDHOs n=9 organoids; unpaired t-test, *p=0.03). **d,** Seahorse energy map of normal and diabetic-like organoids (value = mean ± s.d, n=6). **e,** Number of Mitochondria per μm$^2$ as seen in TEM images of NHOs and PGDHOs (value = mean ± s.d., n=6, two-tailed, unpaired t-test, **p=0.0024). **f,** percentage area of MYL2$^+$ and MYL7$^+$ regions in NHOs and PGDHOs showing ventricular and atrial cardiomyocytes markers, respectively (value = mean ± s.d., n=4, 2-way ANOVA Sidak's multiple comparisons test). *p=0.012, ****p<0.0001, otherwise ns: no significance). BPM: beats per minute, ECAR: extracellular acidification rate, NHOs: normal heart organoids, OCR: oxygen consumption rate, PGDHOs: pregestational diabetes heart organoids. Source data are provided as a Source Data file.

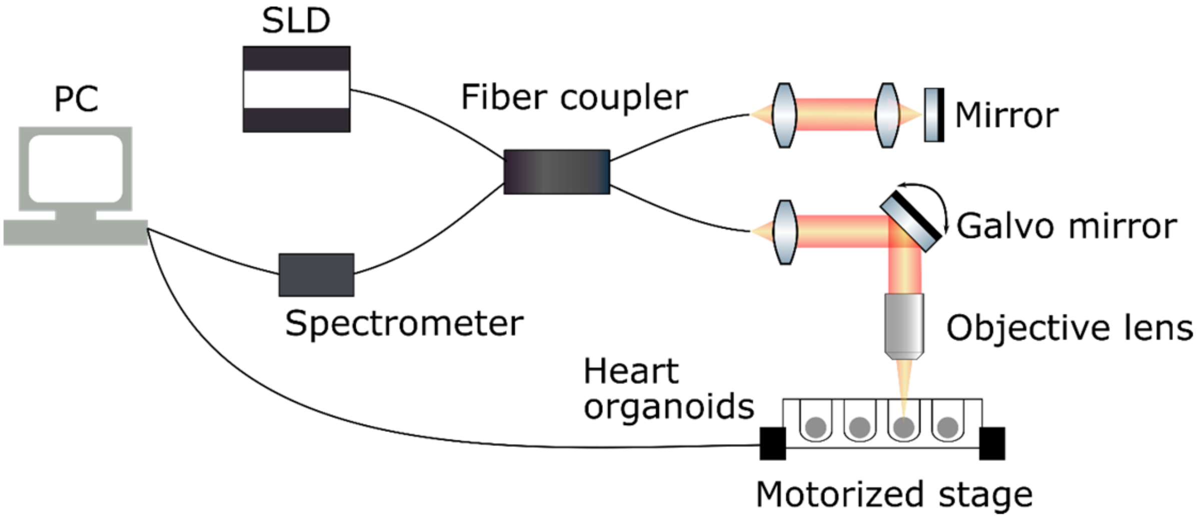


**Supplementary Figure 9.** Illustration of a custom spectral-domain Optical Coherence Tomography imaging system, PC: personal computer, SLD: superluminescent diode.

**Supplementary Table 1** Antibodies used for immunofluorescence in this report.

| | Antibody name | Host Species | Dilution | Catalogue Number | Vendor |
|---|---|---|---|---|---|
| Primary | cTnT (TNNT2) | Mouse | 1:200 | ab8295 | Abcam |
| | WT1 | Rabbit | 1:200 | ab89901 | Abcam |
| | ZO1 (TJP1) | Goat | 1:250 | PA5-19090 | Thermo Fisher Scientific |
| | HAND1 | Rabbit | 1:200 | ab196622 | Abcam |
| | HAND2 | Rabbit | 1:200 | ab200040 | Abcam |
| | MLC2V (MYL2) | Rabbit | 1:200 | ab79935 | Abcam |
| | MLC2A (MYL7) | Mouse | 1:200 | 311-011 | Synaptic Systems |
| | Vimentin (VIM) | Goat | 1:200 | ab11256 | Abcam |
| | CD90/Thy1 | Rabbit | 1:200 | ab133350 | Abcam |
| | NFAT2 (NFATC1) | Rabbit | 1:200 | ab25916 | Abcam |
| | CD31 (PECAM1) | Rabbit | 1:50 | ab28364 | Abcam |
| | COL1A1 | Mouse | 1:200 | M-38 | DSHB |
| | COL4A1 | Mouse | 1:200 | M3F7 | DSHB |
| | FBN1 | Mouse | 1:200 | CPTC-FBN1-1 | DSHB |
| Secondary | Alexa Fluor 488 | Donkey anti-mouse | 1:200 | A-21202 | Thermo Fisher Scientific |
| | Alexa Fluor 488 | Donkey anti-rabbit | 1:200 | A-21206 | Thermo Fisher Scientific |
| | Alexa Fluor 594 | Donkey anti-mouse | 1:200 | A-21203 | Thermo Fisher Scientific |
| | Alexa Fluor 594 | Donkey anti-rabbit | 1:200 | A-21207 | Thermo Fisher Scientific |
| | Alexa Fluor 647 | Donkey anti-goat | 1:200 | A32849 | Thermo Fisher Scientific |
