# Supplementary material for: Self-assembling human heart organoids for the modeling of cardiac development and congenital heart disease
Source: Nat Commun. 2021 Aug 26;12:5142. doi: 10.1038/s41467-021-25329-5 (PMC8390749; doi:10.1038/s41467-021-25329-5)
Supplement: Supplementary file 3 — Description of Additional Supplementary Files [file 41467_2021_25329_MOESM3_ESM.docx]

**Description of Additional Supplementary Files**

**Title: Supplementary Movie 1**

Description: Live imaging of representative organoid derived from iPSC-L1 at day 15 of differentiation. Scale bar: 500 µm.

**Title: Supplementary Movie 2**

Description: Live imaging of representative organoid derived from hESC H9 at day 15 of differentiation. Scale bar: 500 µm.

**Title: Supplementary Movie 3**

Description: Live imaging of representative organoid derived from iPSC-L1 after 8 weeks in culture. Scale bar: 500 µm.

**Title: Supplementary Movie 4**

Description: 3D reconstruction of confocal immunofluorescence Z-stack at 60X magnification of PECAM1+ endothelial tissue (green) adjacent and embedded into TNNT2+ myocardial tissue (red), within day 15 hHOs. Scale bar: 50 µm.

**Title: Supplementary Movie 5**

Description: Confocal immunofluorescence Z-stack of a day 15 hHO stained for DAPI (blue), TNNT2 (red) and PEACM1 (green), showing a robust vascular network throughout the organoid. Scale bar: 500 µm.

**Title: Supplementary Movie 6**

Description: High magnification live imaging of representative organoid derived from iPSC-L1 at day 15 of differentiation stained with India ink for 1 hour to enhance contrast. Scale bar: 100 µm.

**Title: Supplementary Movie 7**

Description: OCT imaging cross-sectional view of hHO showing interconnected chambers near the center of the organoid. Scale bar: 200 µm.

**Title: Supplementary Movie 8**

Description: OCT imaging en face view of hHO showing interconnected chambers near the center of the organoid. Scale bar: 200 µm.

**Title: Supplementary Movie 9**

Description: 3D rendering of hHO OCT imaging cross-sectional view showing interconnected chambers near the center of the organoid. Scale bar: 500 µm.

**Title: Supplementary Movie 10**

Description: Cross-sectional view and 3D rendering of chambers in representative hHO. Scale bar: 500 µm.

**Title: Supplementary Movie 11**

Description: Day 14 organoid derived from iPSCs expressing calcium indicator GCaMP6f, showing calcium transients under fluorescence microscope. Scale bar: 500 µm.

**Title: Supplementary Dataset 1**

Description: Gene ontology of RNA Sequencing data
