# Supplementary material for: Self-assembling human heart organoids for the modeling of cardiac development and congenital heart disease
Source: Nat Commun. 2021 Aug 26;12:5142. doi: 10.1038/s41467-021-25329-5 (PMC8390749; doi:10.1038/s41467-021-25329-5)
Supplement: Supplementary file 16 — Reporting Summary [file 41467_2021_25329_MOESM16_ESM.pdf]

nature portfolio

Corresponding author(s): Aitor Aguirre

Last updated by author(s): Jul 23, 2021

Reporting Summary

Nature Portfolio wishes to improve the reproducibility of the work that we publish. This form provides structure for consistency and transparency in reporting. For further information on Nature Portfolio policies, see our Editorial Policies and the Editorial Policy Checklist.

Statistics

For all statistical analyses, confirm that the following items are present in the figure legend, table legend, main text, or Methods section.

n/a	Confirmed	
☐	☒	The exact sample size (*n*) for each experimental group/condition, given as a discrete number and unit of measurement
☐	☒	A statement on whether measurements were taken from distinct samples or whether the same sample was measured repeatedly
☐	☒	The statistical test(s) used AND whether they are one- or two-sided<br>*Only common tests should be described solely by name; describe more complex techniques in the Methods section.*
☒	☐	A description of all covariates tested
☐	☒	A description of any assumptions or corrections, such as tests of normality and adjustment for multiple comparisons
☐	☒	A full description of the statistical parameters including central tendency (e.g. means) or other basic estimates (e.g. regression coefficient) AND variation (e.g. standard deviation) or associated estimates of uncertainty (e.g. confidence intervals)
☐	☒	For null hypothesis testing, the test statistic (e.g. *F*, *t*, *r*) with confidence intervals, effect sizes, degrees of freedom and *P* value noted<br>*Give P values as exact values whenever suitable.*
☒	☐	For Bayesian analysis, information on the choice of priors and Markov chain Monte Carlo settings
☒	☐	For hierarchical and complex designs, identification of the appropriate level for tests and full reporting of outcomes
☒	☐	Estimates of effect sizes (e.g. Cohen's *d*, Pearson's *r*), indicating how they were calculated

Our web collection on statistics for biologists contains articles on many of the points above.

Software and code

Policy information about availability of computer code

Data collection	For visualization and bioinformatics: Microsoft Excel 365 v2106, R 3.4.1, Graphpad Prism 8, Phantasus v1.11.0, ENRICHR (https://maayanlab.cloud/Enrichr/)<br>For image processing and visualization: MATLAB R2016a, LAS-AF-Lite 2.6.0
Data analysis	For analysis of statistics and bioinformatics: Microsoft Excel 365 v2106, R 3.4.1, Graphpad Prism 8<br>For image analysis and visualization: ImageJ 1.50d, MATLAB R2016a<br>For RNA-Seq: Phantasus v1.11.0, ENRICHR (https://maayanlab.cloud/Enrichr/), edgeR v3.34.0, DESeq2 v1.18.1, HTSeq v0.13.5, ToppGene Suite (http://toppgene.cchmc.org)

For manuscripts utilizing custom algorithms or software that are central to the research but not yet described in published literature, software must be made available to editors and reviewers. We strongly encourage code deposition in a community repository (e.g. GitHub). See the Nature Portfolio guidelines for submitting code & software for further information.

Data

Policy information about availability of data

All manuscripts must include a data availability statement. This statement should provide the following information, where applicable:

- Accession codes, unique identifiers, or web links for publicly available datasets
- A description of any restrictions on data availability
- For clinical datasets or third party data, please ensure that the statement adheres to our policy

All organoid data sets shown in this study are available at the National Center for Biotechnology Information Gene Expression Omnibus repository under accession number GSE153185. RNA-Seq data from monolayer differentiation method 2 and fetal hearts were obtained from GSE106690. All other data generated and/or

analysed during this study is included in the published article and its supplementary information files.

Field-specific reporting

Please select the one below that is the best fit for your research. If you are not sure, read the appropriate sections before making your selection.

☒ Life sciences ☐ Behavioural & social sciences ☐ Ecological, evolutionary & environmental sciences

For a reference copy of the document with all sections, see nature.com/documents/nr-reporting-summary-flat.pdf

Life sciences study design

All studies must disclose on these points even when the disclosure is negative.

Sample size	Power analyses of sample size were not done a priori. The rationale for pooling n=10 organoids for RNA-seq was based on a common and resource efficient strategy that also provides robust averaged RNA-Seq data and is common across relevant publications (Kim et. al., 2019, PNAS, PMID: 31072937, Xiang et. al., 2017, Cell Stem Cell, PMID: 28757360. For all other experiments, n=5-12 were used under most circumstances (unless otherwise stated in the figure legend) where independent experiments are used in accordance with common practice in the field and relevant publications cited in this study (Lee et. al., 2020, Nature Communications, Richards et. al., 2017, Biomaterials, Andersen et. al., 2018, Nature Communications).
Data exclusions	No data were excluded.
Replication	Replications are mentioned in figure legends and the Statistics and Reproducibility section of the Methods, most experiments represent n=5-12 independent organoids. Technical and experimental replicates performed are noted in figure legends. Several different cell lines were also used to support reproducibility.
Randomization	When multiple organoids were available, they were always randomly selected for experiments according to treatment or condition.
Blinding	In the generation of human heart organoids, the differentiation of EBs from human PSCs with different conditions and treatments was performed in a non-blinded manner. Image acquisition was performed with consistent parameters, and cell counting was performed with consistent threshold settings and no data was excluded to limit investigator bias.

Reporting for specific materials, systems and methods

We require information from authors about some types of materials, experimental systems and methods used in many studies. Here, indicate whether each material, system or method listed is relevant to your study. If you are not sure if a list item applies to your research, read the appropriate section before selecting a response.

Materials & experimental systems

n/a	Involved in the study	
☐	☒	Antibodies
☐	☒	Eukaryotic cell lines
☒	☐	Palaeontology and archaeology
☒	☐	Animals and other organisms
☒	☐	Human research participants
☒	☐	Clinical data
☒	☐	Dual use research of concern

Methods

n/a	Involved in the study	
☒	☐	ChIP-seq
☒	☐	Flow cytometry
☒	☐	MRI-based neuroimaging

Antibodies

Antibodies used	cTnT: Anti-Cardiac Troponin T antibody [1C11] (ab8295), Abcam.<br>WT1: Recombinant Anti-Wilms Tumor Protein antibody [CAN-R9(IHC)-56-2] (ab89901), Abcam. Protein.<br>ZO1 (TJP1): ZO-1 Antibody (PA5-19090), ThermoFisher Scientific.<br>HAND1: Anti-HAND1 antibody (ab196622), Abcam.<br>HAND2: Recombinant Anti-HAND2 antibody [EPR19451] (ab200040), Abcam.<br>MLC2V (MYL2): Anti-Myosin Light Chain 2 antibody (ab79935), Abcam.<br>MLC2A (MYL7): MLC-2A - 311 011, Synaptic Systems.<br>Vimentin (VIM): Anti-Vimentin antibody (ab11256), Abcam.<br>CD90/Thy1: Recombinant Anti-CD90 / Thy1 antibody [EPR3133] (ab133350), Abcam.<br>NFAT2 (NFATC1): Anti-NFAT2 antibody (ab25916), Abcam.<br>CD31 (PECAM1): Anti-CD31 antibody (ab28364), Abcam.<br>COL1A1: collagen(pro-) type I (M-38), DSHB.<br>COL4A1: Collagen, type IV, alpha 1/ collagen, type IV alpha 2 (M3F7), DSHB.<br>FBN1: Fibrillin 1 Peptide 1 (CPTC-FBN1-1), DSHB.

	Alexa Fluor 488: Donkey anti-Mouse IgG (H+L) Highly Cross-Adsorbed Secondary Antibody, Alexa Fluor 488 (A-21202), ThermoFisher Scientific.<br>Alexa Fluor 488: Donkey anti-Rabbit IgG (H+L) Highly Cross-Adsorbed Secondary Antibody, Alexa Fluor 488 (A-21206), ThermoFisher Scientific.<br>Alexa Fluor 594: Donkey anti-Mouse IgG (H+L) Highly Cross-Adsorbed Secondary Antibody, Alexa Fluor 594 (A-21203), ThermoFisher Scientific.<br>Alexa Fluor 594: Donkey anti-Rabbit IgG (H+L) Highly Cross-Adsorbed Secondary Antibody, Alexa Fluor 594 (A-21207), ThermoFisher Scientific.<br>Alexa Fluor 647: Donkey anti-Goat IgG (H+L) Highly Cross-Adsorbed Secondary Antibody, Alexa Fluor Plus 647 (A32849), ThermoFisher Scientific.
Validation	cTnT: Suitable for: ICC, IHC-P, Sandwich ELISA. Validated for detecting Troponin T in human cardiac muscle.<br>WT1: Suitable for: WB, IHC-P, Flow Cyt (Intra), ICC/IF. Validated for detecting human Wilms Tumor Protein.<br>ZO1 (TJP1): Suitable for: Immunocytochemistry (ICC/IF). Validated for detecting the internal amino acids of TJP1 in humans.<br>HAND1: Suitable for: ICC/IF. Validated for detecting Human HAND1.<br>HAND2: Suitable for: IHC-P, WB, ICC/IF, IP, Flow Cyt (Intra). Validated for detecting Human HAND2 in the heart.<br>MLC2V (MYL2): Suitable for: WB, IHC-P, IP. Validated for detecting Human Myosin Light Chain 2.<br>MLC2A (MYL7): Suitable for: WB, ICC, IHC, IHC-P/FFPE. Validated for detecting recombinant protein corresponding to AA 1 to 175 from human MLC-2A.<br>Vimentin (VIM): Suitable for: IHC-P. Validated for detecting tissue, cells or virus corresponding to Human Vimentin.<br>CD90/Thy1: Suitable for: IHC-P, WB, ICC. Validated for detecting human CD90/Thy1.<br>NFAT2 (NFATC1): Validated for detecting Human NFATc1.<br>CD31 (PECAM1): Suitable for: IHC-P. Validated for detecting Human CD31.<br>COL1A1: Suitable for: ELISA, FACS, FFPE, Immunofluorescence, Immunohistochemistry, Immunoprecipitation, Western Blot. Validated for detecting Human Carboxyterminal propeptide type I collagen.<br>COL4A1Suitable for: ELISA, FFPE, Immunofluorescence, Immunohistochemistry, Immunoprecipitation, Western Blot. Validated for detecting Human type IV collagen.<br>FBN1: Validated for detecting Human FBN1.<br>Alexa Fluor 488: Suitable for: Immunohistochemistry (IHC), Immunocytochemistry (ICC/IF). Validated for detecting mouse tissue and antibodies.<br>Alexa Fluor 488: Suitable for: Immunohistochemistry (IHC), Immunocytochemistry (ICC/IF), Flow Cytometry (Flow). Validated for detecting rabbit tissue and antibodies.<br>Alexa Fluor 594: Suitable for: Immunohistochemistry (IHC), Immunocytochemistry (ICC/IF). Validated for detecting mouse tissue and antibodies.<br>Alexa Fluor 594: Suitable for: Immunohistochemistry (IHC), Immunohistochemistry (Frozen) (IHC (F)), Immunocytochemistry (ICC/IF), Flow Cytometry (Flow). Validated for detecting rabbit tissue and antibodies.<br>Alexa Fluor 647: Suitable for: Western Blot (WB), Immunocytochemistry (ICC/IF). Validated for detecting goat tissue and antibodies.

Eukaryotic cell lines

Policy information about cell lines

Cell line source(s)	Cell lines were purchased except for iPSC-L1 which was developed in house, and iPSC GCaMP6f which was given as a gift from the Huebsch's lab (Huebsch et. al. 2015).<br>AICS-0037-172 (Coriell Institute for Medical Research, alias AICS)<br>iPSCORE_16_3 (WiCell, alias iPSC-16; UCSD013i-16-3)<br>H9 (WiCell, WA09)<br>HEK293T (ATCC)
Authentication	Purchased cells were authenticated by vendors, typically by STR profiling. For in-house and gifted cells lines, a battery of common iPSC tests was used, including pluripotency testing by using pluripotency marker expression by IF and FACS, karotyping to confirm genomic integrity and teratoma formation.
Mycoplasma contamination	Mycoplasma testing was performed routinely in the lab. All cell lines tested negative.
Commonly misidentified lines<br>(See ICLAC register)	No commonly misidentified cell lines were employed.
